# The Case for Authentic Student Assessment in Distance Digital Pharmacy Education

**DOI:** 10.15694/mep.2021.000006.1

**Published:** 2021-01-11

**Authors:** Amjad Qandil, Ruba Darweesh, Abeer Al-Ghananeem

**Affiliations:** 1 Commission for Academic Accreditation; 2 Jordan University of Science and Technology; 3 Sullivan University

**Keywords:** Digital Education, Authentic Assessment, Pharmacy Education, Distance Learning, e-Learning' Online Assessment

## Abstract

This article was migrated. The article was marked as recommended.

Distance online assessment is facing serious challenges. This opinion piece discusses these challenges and focuses on potential authentic assessment methods that can be used effectively in distance digital pharmacy education.

The authentic assessment methods discussed are case studies, role-play, discussion panels and debates, student e-portfolios, computer-based simulation, diagrams and graphical presentations. These methods allow assessment to drive learning by performing tasks that are related to real professional situations and challenges followed by quantitative and qualitative feedback. Authentic assessment will require the development of clear rubrics to maintain transparency, consistency and fair evaluation of student achievement.

The implementation of online assessment involves some challenges such as the level of institutional, faculty and student’s technological capacity. Financial burden on low-to-medium income families and some institutions, meaningful training on the software and hardware, and students’ compliance with the code of ethics and academic honesty are major issues. That said, well-planned authentic assessment tools can overcome many of these challenges.

As these methods amass momentum, more creativity and innovation in assessment will be introduced, enriching and facilitating digital pharmacy education. The author calls for utilizing more formative feedback and reflection methods, focusing on demonstrating learning and professional competencies rather than on superficial letter grades.

## Introduction

It would not be an overstatement to say that COVID-19 is a die-or-evolve “extinction event” that will be a major factor in the current development of the world and will forever transform teaching and learning. This crisis revealed that online dissemination of knowledge is relatively easy, and that the majority of students are capable of finding, processing and using online knowledge. On the other hand, a major gap was exposed in the design, deployment and management of distance online student. This topic is becoming a central point of interest in digital education. Traditionally, tests and examinations require invigilation to guarantee the integrity of the process and to safeguard against cheating. This process becomes more complicated when done online, as the elaborate solutions for distance invigilation are costly and require special setup by the students. Furthermore, the transition to online education could be a turning point for higher education institutions to minimize the dependence on on-site examinations. One way to realize this is by maximizing the use of authentic assessment methods to measure students’ achievement of the learning outcomes related to the application of knowledge and skills. Additionally, many authentic assessment tools can help in evaluating students’ attainment of the effective domain learning outcomes. With proper planning, training and execution, this transition can be smooth and pleasantly efficient once put into effect.

Authentic assessment is defined as “...engaging and worthy problems or questions of importance, in which students must use knowledge to fashion performances effectively and creatively. The tasks are either replicas of or analogous to the kinds of problems faced by adult citizens and consumers or professionals in the field.”( Wiggins, 1993). Whereas traditional assessment may provide direct evidence of what a student knows, it is lacking in providing direct evidence of what the student can do. On the other hand, authentic assessment provides direct evidence of both what the student knows and can do. Online assessment in patient-oriented pharmacy is facilitated by the fact that the practice is intensively reliant on cognitive and social skills; with the contribution of few psychomotor skills related to pharmacy compounding, use of self-care devices (e.g. inhalers and nebulizers) and basic patient physical examination (e.g. sphygmomanometers).

This opinion piece will mainly focus on authentic assessment methods that follow a constructivist approach that combines learning with assessment, i.e. that facilitate learning by assessment. Additionally, this opinion piece will have a social collaborative dimension to it. In this integrative mode, especially when in a digital environment, the instructor needs to act as a facilitator who shares ownership of learning with students and accepts to share control of student learning ( Cochrane, 2012; Flavin, 2012). In fact, students are already collaborating and taking some responsibility for their learning, mostly through parallel “digital communities”, to supplement the formal learning communities led by instructors ( Barber, King and Buchanan, 2015). The authors may not introduce new assessment methods, but they will bring the attention of pharmacy educators to the ability to adopt and adapt authentic assessment tools to the virtual learning environment.

## Case studies

Simulated patient cases have been shown to be valid tools for the assessment of clinical pharmacy skills ( Rudall *et al.*, 2015). Where an MCQ or short essay examination can show if students have the required knowledge to construct the medication care plan, an authentic assessment by presenting the student with a real-life or simulated mock scenario for a patient with heart failure will demonstrate the ability of the student to design the medication care plan and at which level of competence. Furthermore, different levels of complexity can be achieved with case-based assessment, as it can be escalated by adding comorbidities and drug-drug interactions. Working on these case studies will not need invigilation as students are allowed to utilize available resources and literature. Furthermore, case-based assessment can be a collaborative exercise and it lends itself very well to interprofessional education using video conferencing platforms. Simulated cases can incorporate integrated challenges in which various skills and knowledge from different professions need to be coordinated to achieve an accepted conclusion. This approach can be used in the assessment of therapeutics, applied therapeutics, pharmacokinetics, medication therapy management (MTM) and over the counter (OTC) medications courses online without the need of face-to-face interaction. Online discussions between skilled instructors and students can easily reveal if the student has designed the plan him/herself.

The achievement of pharmacy clinical and experiential training learning outcomes and competencies by students requires significant face-to-face interaction with patients and a physical presence in care settings. Nevertheless, current authentic assessment methods can be easily performed online and from a distance. For example, the discussion of patient cases with peers and preceptors can be easily realized in a virtual environment. It is likely in the near future that a pharmacist will be able to take patient history in inpatient settings virtually. This arrangement will most likely be accelerated as it increases the margin of healthcare providers’ safety in times of epidemics and pandemics. This strengthens the case for training and assessing pharmacy students on virtual patient care practices.

Pharmaceutical sciences can use case-based assessment to measure student’s abilities to analyze medication errors, explain structure-activity relationships, examine pharmacogenetic findings and predict their effect on medication choice and care decisions ( Stewart, Buckner and Wildfong, 2011; Abdel-Halim, 2020b).

## Role Play, Discussion Panels and Debates

Verbal assessment such as role-play, discussion panels and debates which integrate learning with assessment can be deployed online. Topics in pharmacy law and health and research ethics can be discussed in online debates moderated by faculty members. In addition to being a learning activity, these debates can provide authentic assessment of the students’ grasp of regulatory and ethical issues and their ability to evaluate and give decisions in complex ethical situations. Furthermore, role-play, which combines play, games and simulation, can be an enjoyable experience for both the instructor/facilitator and the students while preserving learning benefits through reflections guided by the facilitator ( McSharry and Jones, 2000). Role-play offers an opportunity to design a creative approach to problem solving that can be done virtually to enrich student learning. Role-play can be particularly valuable for the development and assessment of communication skills including, but not limited to conflict management and dealing with healthcare professionals, patients and caregivers. Well-crafted role-play scenarios can be used to assess a student’s ability to offer counseling to simulated patients and caregivers. These scenarios can be also be incorporated into a virtual OSCE.

The authors believe that these types of integrated learning and assessment activities should be mandatory and be intentionally designed to train students on the provision of healthcare online, such as in telehealth and virtual health visits which have been deployed in various healthcare settings around the globe to mitigate the effects on COVID-19 on the healthcare system.

## Student e-Portfolios

The use of student e-portfolios is a powerful tool to engage students in self-assessment of their cognitive and social learning across an educational program, or parts of it ( Giering and Firdyiwek, 2020). A student e-portfolio can be designed differently depending on its purpose in the curriculum. It can showcase a student’s past work to provide a timeline for the pace, quantity and quality of a student’s growth. This timeline is made richer by a student’s meaningful reflection and self-assessment of his/her learning. Students believe that e-portfolios can enhance authentic assessment, improve problem solving skill and critical thinking and promote creativity ( Koraneekij and Khlaisang, 2019). e-Portfolios can be formatively assessed by mentors and preceptors or summatively to provide grades. Work added to an e-portfolio can include recordings of student presentations, OSCE stations and patient counseling role-play. This will provide insight on the iterative and continuous improvement in students’ communication skills and competence. Mentors and preceptors can review these e-portfolios periodically to provide guidance and feedback to students, to reinforce good practices, and/or provide recommendations for improvement. Student feedback and discussion of performance can be done virtually via online meetings.

## Computer-based Simulation

The use of computer simulations for learning and assessment in community, inpatient and outpatient pharmacy experiential training settings is gaining momentum. This type of virtual learning environments will only become better as more people use them, especially if users are able to add to the scenarios, as well as by incorporating artificial intelligence (Imitated Environments, Accessed May 18, 2020). Assessment in some of these platforms is built-in and is performed in real time by the system.

With many free 3D-visualization tools available, students can learn about drug-receptor interactions in a virtual three-dimensional space that shows atoms, bonds, shapes and stereochemical arrangements ( Abdel-Halim, 2020a). The visualization software are available online, and can be used by students remotely, allowing synchronous or self-paced asynchronous interactive learning. The same virtual environment can be used to assess students, which allows for different levels of complexity by introducing chemical variations to drugs and asking students to explain their potential effect on affinity and efficacy.

## Diagrams and Graphical Presentations

Analysis of the mechanisms of drug action, drug pharmacokinetics, and pharmaceutical processes can be assessed by visual tools such as diagrams drawn by students. The complexity of the assessment can be increased by introducing disruptive event(s) in one or more of the steps in the diagram and requiring the students to predict the outcome and justify their prediction. In addition to diagrams, transforming data into graphs with appropriate levels of written commentary and conclusions is suitable to assess skills in pharmacokinetics, pharmaceutics and biopharmaceutics. There is a plethora of software and online solutions that can be made available to students to produce their diagrams and graphs. But if access to these tools is not possible or is overly burdensome, students can use pens, paper (plain or graph paper) and rulers to produce diagrams, then scan their products using mobile devices or the laptop cameras to share online. An accessible software solution is using simulation Microsoft Excel sheets in pharmacokinetics ( Wu and Hu, 2011). These sheets can be easily used to simulate changes in different parameters and characterize the effects on the related pharmacokinetic processes.

## Rubrics

Using authentic assessment of student achievement will require the design of suitable rubrics based on the intended learning outcomes and competencies. These rubrics will need to provide clear criteria for success. Each criterion will include producing the correct product as well as asking students for justifications to their decisions. Rubrics will be announced and explained to students beforehand to allow them to understand the task’s expectations. Graders should be trained on the use of these rubrics to reduce the bias brought by the graders’ perceptions and reduce inter-student and inter-grader grade variation to maintain the integrity of student evaluation practices.

## Programmatic Assessment

Authentic assessment methods lend itself very well to the programmatic assessment approach where decisions to pass or fail are “decoupled from individual assessment moments” ( van der Vleuten *et al.*, 2015). Adopting such approach requires no less than a paradigm shift in how education programs view student assessment and progression decisions. One of the most interesting concepts in programmatic assessment is that it amalgamates formative and summative assessment into a range of low- to high-stakes decisions. This particularly important as authentic assessment provide amble opportunity for formative feedback. Another equally interesting concept of this approach is the longitudinal assessment of competencies. The development of competencies such as collaboration, professionalism, and communication are essentially longitudinal. Information on a certain competency can be tracked and aggregated across multiple assessment tools if a master plan for assessment was developed based on a competency framework ( van der Vleuten *et al.*, 2015). The development of such competencies can be measured by authentic assessment methods that mimic real life problems under unstandardized conditions.

## Challenges

Online distance learning and distance assessment of student achievement does not come without challenges. Firstly, the digital capacity of institutions, faculty and students, that is required to shift partially or completely to a digital environment will be challenging. Digital capacity spans multiple areas including, development of an institutional digital transformation policy, building digital material vs. digitizing analog ones, making all processes interconnected, internet connectivity and bandwidth, suitability and sustainability of hardware and software, upskilling current personnel and recruiting new talent, appropriate literacy and training of all parties on digital education processes, and the development of off-campus digital environment. Accessibility and/or poor internet connectivity are major barriers of distance learning in medical education ( O’Doherty *et al.*, 2018), with a major impact on distance online assessment. The parts of authentic assessment methods that need no videoconferencing, such as submission of portfolios and uploading assignments will not suffer significantly because of poor connectivity. If students have no access to suitable communication devices, this will result in inconsistent engagement and force students to miss learning and assessment sessions. Although in many cases a smartphone will be enough, securing more expensive devices such as tablets or laptops will impose extra financial burden on families, leading to potential disparity that may need governmental and institutional intervention ( Qandil and Abdel-Halim, 2020). The infrastructure needed for distance learning and assessment includes software, i.e. a suitable Learning Management System (LMS) and an assessment platform, which can provide sufficient tools to manage assessment, and hardware (servers) which incur financial consequences on the institutions introducing burden to institutions in low-to-medium income countries ( Frehywot *et al.*, 2013). The use of available software and hardware requires robust training of students, faculty and staff, including IT and educational technologists. This technical training, through workshops and courses, is crucial to implement the available distance assessment tools. Lack of suitable technical education literacy is currently a main problem in deploying effective distance learning. Additionally, real-time IT support and LMS support with open channels of communication between students, faculty members, the support team are critical in maintaining online learning assessment. The provision of training and support services, especially at the beginning, may be overwhelming to institutions and their personnel. In the long run, this will require the recruitment of professionals dedicated to digital education content creation and provision of technical support.

Assuring students’ compliance with the code of ethics, identity verification and academic honesty is still a challenge. Assessment tools utilizing MCQs, even those that focus on higher learning skills are high on the spectrum of security demands. As mentioned earlier, a strength of authentic assessment methods is that invigilation is not needed because student achievement can be verified during online discussions, and potential plagiarism can be checked using a range of online tools including some free options.

Large class sizes can pose a challenge to grading intensive authentic assessment methods such as numerical assignments, reports and portfolios. This might be alleviated by recruiting teaching assistants although they will need extensive training. On the other hand, the online platform offers an opportunity to employ authentic assessment methods that involve collaborative and interprofessional case discussions, debates, role play and panel, for large classes by overcoming the need for a physical space at institutions to run these activities. These challenges can create resistance and/or negative attitude toward online assessment among instructors and students. Although the COVID-19 pandemic exacerbated it, it also led to genuine and practical efforts to overcome them.

## Implications

While maximizing the utilization authentic assessment methods provides a formidable solution for the challenges of digital online education, It also improves the evaluation decisions of any pharmacy program regardless of it mode of delivery. It allows students to perform meaningful tasks that are drawn from real professional challenges that students, when graduated, should be capable of answering. As faculty and students become more familiar and comfortable using these methods, their creativity will flourish. As more innovative tools emerge, pharmacy education will be enriched, and real measures of student achievement of the intended learning outcomes and graduate level of competencies will be developed further.

Finally, all the assessment methods in this opinion piece are largely discussed in light of summative practices. Nevertheless, the authors urge pharmacy educators to reconsider their assessment convictions and to allow more space for formative feedback in assessing student achievement. A well-thought deliberately designed program that includes summative and formative assessment will go a long way in driving learning by assessment ( van der Vleuten *et al.*, 2012).

## Conclusions

Authentic student assessment will allow students to focus on learning the concepts crucial to their profession and demonstrate competence as opposed to becoming fixated on the fallacy of grades.

## Take Home Messages

• Authentic assessment provides a formidable alternative to high stake MCQ-based assessment in distance online learning
• Authentic assessment can be used as an assessment for learning and an assessment of learning
• Authentic assessment can provide meaningful evaluation of student achievements

## Notes On Contributors

Prof. Amjad M. Qandil is currently a Commissioner and an education expert at the Commission for Academic Accreditation in the United Arab Emirates. Prior to his current role, he spent more than 20 years teaching undergraduate and postgraduate medicinal chemistry and leading a research laboratory in medicinal chemistry and drug design in addition to engaging in pharmacy education research. Prof. Qandil holds a PhD from Purdue University, USA. ORCID ID: https://orcid.org/0000-0003-3269-0678

Dr. Ruba S. Darweesh is an Assistant Professor of Clinical Pharmacokinetics at the Department of Pharmaceutical Technology in Faculty of Pharmacy, Jordan University of Science and Technology. In addition to teaching, she is leading a research group in Pharmacokinetics and Biopharmaceutics. Dr. Darweesh holds a PhD from Virginia Commonwealth University, USA.

Prof. Abeer M. Al-Ghananeem is Professor and Director of Research, College of Pharmacy and Health Sciences, Sullivan University, Louisville Campus. She is an Academic Leadership Fellow of the American Association of Colleges of Pharmacy (AACP) and Commissioner, Chair-Elect for the Accreditation Council for Pharmacy Education (ACPE)’s International Services Program. Prof. Al-Ghananeem holds a PhD from the University of Kentucky in Lexington, Kentucky, USA.
