# Supplementary material for: The Case for Authentic Student Assessment in Distance Digital Pharmacy Education
Source: MedEdPublish (2016). 2021 Jan 11;10:6. [Version 1] doi: 10.15694/mep.2021.000006.1 (PMC10939618; doi:10.15694/mep.2021.000006.1)
Supplement: Peer review history

# Reviewer report: Reviewer response for version 1

**DOI:** 10.21956/mep.18863.r26904

**Published:** 2021-10-09

**Authors:** Anita Samuel

This review has been migrated. The reviewer awarded 4 stars out of 5 This is a practical and informative article that takes lessons learned from remote teaching and presents them as sustainable lessons for the future of pharmacy education. The article is clearly written and easy to follow. The authors provide a holistic review of authentic assessments appropriately noting the need for robust rubrics.The challenge of large classes is not sufficiently addressed beyond the role of teaching assistants. This is something that should be given more consideration as many instructors balk at the workload implications of authentic assessments.

Reviewer Expertise:

NA

No decision status is available

# Reviewer report: Reviewer response for version 1

**DOI:** 10.21956/mep.18863.r26905

**Published:** 2021-01-14

**Authors:** Keith Wilson

**Affiliations:** 1 Dalhousie University

This review has been migrated. The reviewer awarded 4 stars out of 5 The authors present a succinct case for the adoption of authentic student assessment modalities using distance education techniques. The article highlights some common types of assessment that can be used in this emerging virtual education world. Namely, case studies, role play, e-portfolios and others form a menu of assessment techniques that could be widely adopted, even beyond pharmacy education. The authors provide practical consideration of these techniques and highlight many positive aspects that make them accessible in many situations.The merit of the approaches that the authors suggest would be enhanced with a critical exploration of the evidence for each. This would be very beneficial for those needing to find a solution when resources are scarce. In some ways a table that lists challenges and evidence-informed solutions would be very helpful for the reader.This opinion piece is well written and will be of particular interest to a broad audience involved in assessment of learners. The authors are to be commended for highlighting these more authentic techniques of assessment that can be utilized in this new educational world. Readers will take away a number of ideas that can be applied broadly in health professions education.

Reviewer Expertise:

NA

No decision status is available
